# Higher Prostate Weight Is Inversely Associated with Gleason Score Upgrading in Radical Prostatectomy Specimens

**DOI:** 10.1155/2013/710421

**Published:** 2013-10-31

**Authors:** Leonardo Oliveira Reis, Emerson Luis Zani, Leandro L. L. Freitas, Fernandes Denardi, Athanase Billis

**Affiliations:** Departments of Urology and Pathology, Faculty of Medical Sciences, University of Campinas (Unicamp), Rua Tessália Vieira de Camargo 126, Cidade Universitária “Zeferino Vaz,” 13083-887 Campinas-SP, Brazil

## Abstract

*Background*. Protective factors against Gleason upgrading and its impact on outcomes after surgery warrant better definition. *Patients and Methods*. Consecutive 343 patients were categorized at biopsy (BGS) and prostatectomy (PGS) as Gleason score, ≤6, 7, and ≥8; 94 patients (27.4%) had PSA recurrence, mean followup 80.2 months (median 99). Independent predictors of Gleason upgrading (logistic regression) and disease-free survival (DFS) (Kaplan-Meier, log-rank) were determined. *Results*. Gleason discordance was 45.7% (37.32% upgrading and 8.45% downgrading). Upgrading risk decreased by 2.4% for each 1 g of prostate weight increment, while it increased by 10.2% for every 1 ng/mL of PSA, 72.0% for every 0.1 unity of PSA density and was 21 times higher for those with BGS 7. Gleason upgrading showed increased clinical stage (*P* = 0.019), higher tumor extent (*P* = 0.009), extraprostatic extension (*P* = 0.04), positive surgical margins (*P* < 0.001), seminal vesicle invasion (*P* = 0.003), less “insignificant” tumors (*P* < 0.001), and also worse DFS, *χ* ^2^ = 4.28, df = 1, *P* = 0.039. However, when setting the final Gleason score (BGS ≤6 to PGS 7 versus BGS 7 to PGS 7), avoiding allocation bias, DFS impact is not confirmed, *χ* ^2^ = 0.40, df = 1, *P* = 0.530.*Conclusions*. Gleason upgrading is substantial and confers worse outcomes. Prostate weight is inversely related to upgrading and its protective effect warrants further evaluation.

## 1. Introduction

 Gleason score (GS) remains the most widely accepted grading system in the evaluation of prostate cancer and is one of the most important factors influencing tumor prognosis and treatment choice for patients diagnosed with prostate cancer [1]. Nevertheless, several studies have reported a poor Gleason score concordance between biopsy and radical prostatectomy (RP) specimens [1–4].

 Failure of accurately obtaining the biopsy specimen to precisely reflect the true nature of the cancer is especially important for patients considering nonextirpative treatments, such as external beam radiotherapy, brachytherapy, cryotherapy, or expectant management [5].

 Also, whether the clinical outcome of Gleason score discordance is similar to that of concordant tumors of the higher grade, concordant tumors of the lower grade, or somewhere in between remains to be solved.

 Targeting a better guidance to patients during their treatment decision process, we investigated factors predictive of Gleason score upgrading between biopsy and surgical specimens and the impact of discordance scores on postoperative outcomes.

## 2. Materials and Methods

### 2.1. Patient Selection

 A prospectively maintained database of 360 consecutive patients who underwent 10–12 core prostate biopsy and radical prostatectomy at our institution from 1997 to 2009 was reviewed after institutional review board approval. 

Patients who received prior hormone treatment or radiotherapy or refused to authorize the use of their medical records were excluded. 

### 2.2. Pathologic Evaluation

Gleason scores of biopsy and prostatectomy were reanalyzed and regraded by pathological review and categorized as ≤6, 7, and ≥8 by an expert uropathologist (Athanase Billis) according to the 2005 International Society of Urological Pathology (ISUP) Consensus Conference on Gleason Grading of Prostatic Carcinoma [7]. 

Upgrading was considered RP grade in a higher category than the biopsy and downgrading the opposite. After transecting the seminal vesicles at the base, the prostate gland was weighed when fresh after RP, using an electronic scale and its weight was recorded in grams.

The tumor extent was evaluated by a semiquantitative point-count method [6]. Briefly, each quadrant of the whole mount sections of the surgical specimen, which contained eight equidistant points, was drawn on a sheet of paper. During the microscopic examination of the slides, the tumor area was drawn on the correspondent quadrant seen on the paper. The amount of positive points represented an estimate of the tumor extent. More extensive tumors corresponded to >26 positive points and “insignificant” tumors, defined as having volume <0.5 cc and no Gleason grade 4 or 5 component (primary, secondary or tertiary) corresponded approximately to ≤10 positive points [6]. 

### 2.3. Follow-Up Regimen

Evaluated parameters included age, prostate weight, preoperative prostate-specific antigen (PSA) level, PSA density, and tumor extent as continuous variables, and race, biochemical recurrence (BCR), clinical and pathological stages, Gleason grade, extraprostatic extension, positive surgical margins, seminal vesicle invasion, and “insignificant” tumors as categorical variables. 

During the postoperative period, serum PSA was drawn every 3 months during the first year, every 6 months during the second year, and annually thereafter. Total serum PSA was measured using previous validated Immulite PSA kit. PSA ≥0.2 ng/mL after surgery was considered BCR, according to recommendation of the American Urological Association [8]. Patients without evidence of BCR were censored at last followup for disease-free survival (DFS) analyses. 

### 2.4. Statistical Analysis

The chi-square or Fisher's exact test (for expected values less than 5) was used to compare the major categorical variables, the Mann-Whitney test to compare numerical variables between two groups, and Kruskal-Wallis test for comparing numerical variables between three or more groups. The McNemar's test (two categories) and the Bowker's test of symmetry (three categories) were applied to compare the biopsy Gleason score (BGS) and pathological Gleason score (PGS). 

The uni- and multivariate stepwise logistic regression analyses were utilized to study PGS score upgrading predictors. The analysis of Receiver Operating Characteristic (ROC), the area under the curve (AUC), 95% confidence interval, and the levels of sensitivity and specificity were calculated for accurate cut-offs discriminations.

Postoperative disease-free survival was estimated using the Kaplan-Meier method and compared with the log-rank test. A two-sided 5% significance level was adopted for statistical tests (*P* < 0.05).

## 3. Results

 After exclusion criteria, 343 patients met our standards for analysis and the discordance between BGS and PGS was 45.7%.  Table 1 lists patient's demographics in each one of the groups: BGS = PGS (54.23%, *n* = 186), BGS < PGS (37.32%, *n* = 128), and BGS > PGS (8.45%, *n* = 29). 

The mean age of the population was 63.46 (SD = 6.56) years (median 64), and the average weight of all prostates was 40.56 g (median 35; range 11–190). During the mean followup of 80.2 months (median 99), 94 patients (27.4%) had PSA recurrence after radical prostatectomy. Mean pretreatment PSA was 9.63 (SD = 6.72, median = 7.92), range 0.28–51. 

Gleason upgrading led patients to increased clinical stage (*P* = 0.019), more positive points in surgical specimen (*P* = 0.009), extraprostatic extension (*P* = 0.04), positive surgical margins (*P* < 0.001), seminal vesicle invasion (*P* = 0.003), and less “insignificant” tumors (*P* < 0.001). 

Tables 2 and 3 present the results of the uni- and multivariate logistic regression analyses to predict Gleason discordance between biopsy and RP.

According to multivariate logistic regression analysis, lower prostate weight (*P* < 0.001), higher PSA (*P* = 0.003), higher PSA density (*P* < 0.001), and higher BGS (*P* < 0.001) were significantly associated with PGS upgrading. While the upgrading risk decreased 2.4% for each 1 g of prostate weight, it increased 10.2% for every 1 ng/mL of PSA, 72.0% for every 0.1 unity of PSA density, and was 21 times higher for those with BGS 7 (Table 3).

**Figure 1:**
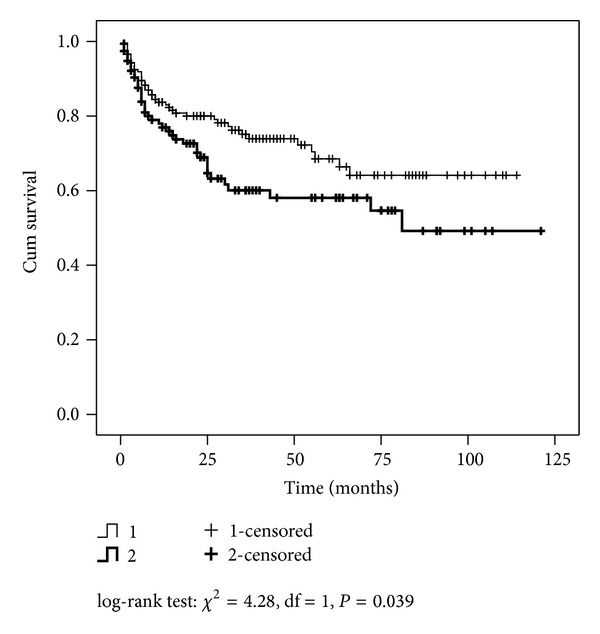
Disease-free survival by Gleason score between biopsy and radical prostatectomy (1: concordant; 2: upgrade).

Patients with Gleason upgrade presented worse disease-free survival compared with concordant Gleason tumors, log-rank test: *χ* ^2^ = 4.28, df = 1, and *P* = 0.039 (Figure 1).

**Figure 2:**
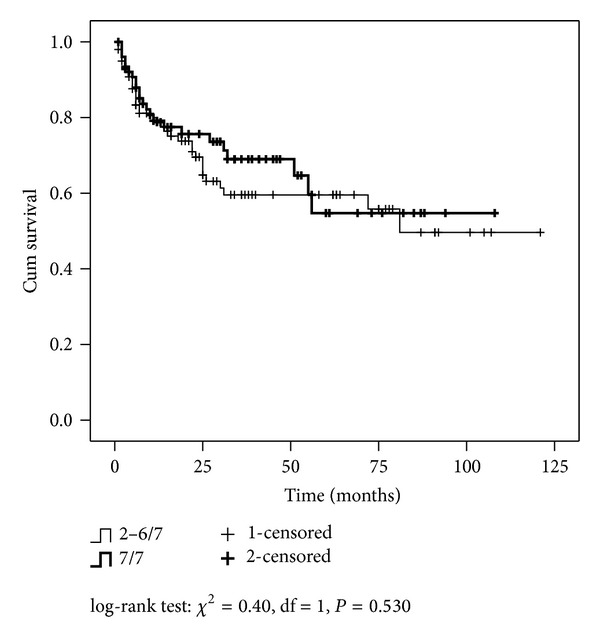
Disease-free survival by Gleason score between biopsy and radical prostatectomy (“2–6” to “7” versus “7” to “7”).

Focusing on PGS 7, comparing PGS 7 that have upgraded (BGS ≤ 6 to PGS 7) with those that was accurately diagnosed on biopsy (BGS 7 to PGS 7), the last were significantly associated with extraprostatic tumor extension (*P* = 0.039), >pT2 pathological stage (*P* = 0.023), and older age (*P* < 0.001). However, disease-free survival was not different, log-rank test: *χ* ^2^ = 0.40, df = 1, and *P* = 0.530, when comparing BGS ≤ 6 to PGS 7 versus BGS 7 to PGS 7, (Figure 2). 

**Figure 3:**
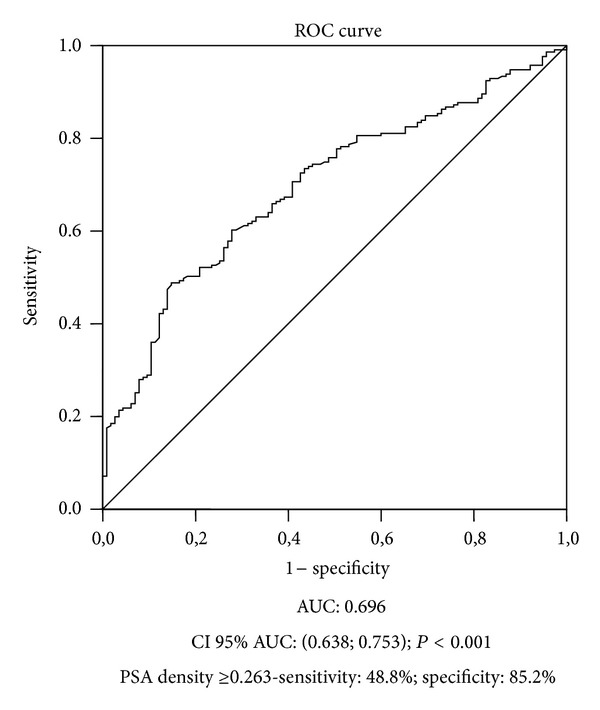
Receiver Operating Characteristic curve analysis for PSA density to preoperatively predict Gleason discordance.

When associating PSA and prostate volume to predict Gleason score upgrading on radical prostatectomy specimens, PSA density ≥ 0.263 significantly discriminated between patients with and without upgrading at surgery (*P* < 0.001), AUC: 0.696, CI 95% 0.638–0.753, sensitivity: 48.8%/specificity: 85.2%, (Figure 3), and also determined disease-free survival, log-rank: *χ* ^2^ = 22.76; GL = 1; *P* < 0.001, (see Supplementary Figure available online at http://dx.doi.org/10.1155/2013/710421).

## 4. Discussion

Gleason score discordance between biopsy and radical prostatectomy specimens is a common finding, with 32%–73% rates reported in the literature [2–4, 9], being more concordant in departments of pathology that regularly evaluate RP specimens (>40 RP specimens annually) [5]. 

Upgrading is the most common problem and downgrading is found in only about 10–15% of cases. In general, adverse findings on needle biopsy accurately predict adverse findings in RP specimen, whereas favorable findings in needle biopsy do not necessarily predict favorable findings in RP specimens in large part due to sampling error, borderline cases, pathology error, intraobserver and interobserver variability [10].

Although prior radical prostatectomy series have shown that patients with a lower BGS experienced significantly better DFS than patients with equal BGS and PGS, suggesting that BGS represents additional prognostic value to PGS [11, 12], in our data while patients with equal BGS and PGS have presented a significant increment of extraprostatic tumor extension (*P* = 0.039), >pT2 pathological stage (*P* = 0.023) and older age (*P* < 0.001) DFS was not different when comparing BGS ≤ 6 to PGS 7 versus BGS 7 to PGS 7.

 Our study is consistent with contemporary data, particularly in the era of PSA and routine 12 core biopsies [13–17], associating Gleason score discordance with adverse pathological features (advanced tumor stage, more positive points in surgical specimen, extraprostatic extension, positive surgical margins, seminal vesicle invasion, and lower rates of “insignificant” tumors) and worse DFS. However, the real independent impact of Gleason upgrading on DFS may be questioned, since when setting the final Gleason score (BGS ≤ 6 to PGS 7 versus BGS 7 to PGS 7), avoiding allocation bias, DFS effect is not confirmed, *χ* ^2^ = 0.40, df = 1, *P* = 0.530, supporting a failure of the initial biopsy to accurately reflect the prostatectomy Gleason score or to add enough prognostic influence that may be applicable to strategies of risk stratification and patient counseling after surgery.

 Together these data support the concept that RP pathological parameters provide an improved prognostic assessment of outcome in men with clinically localized prostate cancer than biopsy parameters [15, 16]. 

 Intriguingly, the multivariate logistic regression analysis showed that prostate weight was a protective factor, decreasing 2.4% upgrading risk for each 1 g of prostate weight, while higher BGS, PSA levels, and PSA density were selected as being significantly associated with further PGS upgrading.

 The protective effect of (higher) prostate weight is an underexplored paradox phenomenon since it is expected that the larger the prostate, the greater the sampling error. 

 Keeping the number of cores around 10 to 12, according to the current optimal technique, the biopsy artifact hypothesis seems to be an insufficient explanation. If sampling error was the central cause of Gleason upgrading, then upgraded tumors would represent larger prostates, smaller tumor burden, or both compared with tumors concordant for the higher grade, strikingly conflicting with our results.

 Among many assumptions, larger glands may produce more PSA due to the presence of benign prostatic hyperplasia, causing a lead-time bias or diagnosis of prostate cancer at an earlier point in the progression of disease, which could justify the protective effect of larger glands regarding upgrading. Otherwise, a large prostate might work as an obstacle to the growth of cancer cells, culminating with less extracapsular extension and consequently less positive surgical margins and lower biochemical recurrence.

 Regardless of the mechanism, it offers the opportunity to accurately predict the final pathological grade based on clinical parameters, improving our ability to inform patients and guide their care. However, it is startling that many prediction tools, such as nomograms, have not taken advantage of the size-weight/grade relationship, neither for surgery nor radiotherapy [18].

 Though there is an association between smaller prostates and Gleason upgrading on uni- and multivariate analysis, aiming to better understand the influence of prostate size, PSA, and Gleason upgrade connection, this study measured the association between PSA and prostate volume once smaller size prostate tends to have a higher PSA density and be more likely to harbor high-grade disease as demonstrated in this study and elsewhere [19, 20]. 

 PSA density adds the mixed impact of both PSA and prostate volume, being also a strong independent predictor of Gleason upgrade. Thus, PSA being an important diagnostic tool, it selects patients for prostate biopsy, inputting PSA related allocation bias. In this scenario, observing that smaller prostates are more likely to have upgraded cancer is somewhat related to the performance characteristics of PSA. We interpret this to mean that when controlling prostate size, PSA is the additional important driver behind upgrading; however, beware of the small prostate once the influence of PSA is subtle.

 The limits of this study are those of any retrospective analysis, the relatively small number of patients, and the lack of overall and disease specific survival, limiting to DFS; however, all prostatectomies was performed at a single institution and a single expert uropathologist reviewed all biopsies and whole-mount RP slides, also detailed morphometric mapping were used to estimate tumor extent and to evaluate margin status, extracapsular extension, or foci of high-grade cancer. Furthermore, this series particularly focuses on the Gleason upgrade issue in real contemporary scenery of PSA and routine 10–12 core biopsies era, utilizing the modified 2005 Gleason system. 

 While the use of final pathological prostate weight should be viewed as a limitation, it has been shown to correlate well with trans-rectal ultrasound prostate volume [21, 22], and both are final pathological Gleason score predictors.

 Lastly, we analyzed prostate weigh, PSA and PSA density as continuous variables, giving complete information in addition to categorical variables in others studies. Also, Gleason score up- and downgrading was considered among more representative classes: ≤6, 7, and ≥7; once between 2 to 6 (lower risk range) and 8 to 10 (higher risk range) there is a recognized less powerful risk stratification.

## 5. Conclusions

 Gleason score discordance between biopsy and radical prostatectomy specimens in prostate cancer patients is substantial and has potential clinical significance in predicting worse oncologic outcomes. 

 Prostate weight is inversely associated with Gleason upgrading in RP specimens and its protective effect warrants further evaluation, focusing on using prostate size in models to predict upgrading and downgrading on final pathology and outcomes.

## Supplementary Material

Supplementary Figure: PSA density (≥ 0.263) determined disease free survival.

## Figures and Tables

**Table 1:** Patients' demographics by Gleason score at biopsy versus radical prostatectomy (RP).

Feature	Gleason score			*P* value
	Biopsy < RP (upgraded)	Biopsy = RP	Biopsy > RP (downgraded)	
Overall	128 (37.32%)	186 (54.23%)	29 (8.45%)	
Age (Median/Min Max)	62.76 (43–76)	63.94 (46–76)	63.55 (46–74)	0.317
Race				0.522
White	98 (76.56%)	156 (83.87%)	24 (82.76%)	
Black	28 (21.88%)	27 (14.52%)	5 (17.24%)	
Yellow	2 (1.56%)	3 (1.61%)	0	
PSA	10.29 (0.28–8.60)	8.95 (0.6–29.7)	11.1 (3.10–44)	0.192
Prostate weight	37.45 (15–94)	42.34 (11–190)	42.30 (15–110)	0.373
PSA density	0.31 (0.06–0.86)	0.25 (0.09–0.66)	0.19 (0.03–0.63)	<0.001
Clinical stage				0.019
T1c	52 (44.44%)	99 (56.25%)	8 (30.77%)	
T2a	46 (39.32%)	58 (32.95%)	10 (38.46%)	
T2b	13 (11.11%)	17 (9.66%)	7 (26.02%)	
T2c	6 (5.13%)	2 (1.14%)	1 (3.85%)	
Biopsy Gleason score				<0.001
≤6	115 (89.84%)	99 (53.23%)	0	
7	13 (10.16%)	84 (45.16%)	19 (65.52%)	
≥8	0	3 (1.61%)	10 (34.48%)	
Pathological Gleason score				<0.001
≤6	0	100 (53.76%)	18 (62.07%)	
7	112 (87.50%)	83 (44.62%)	11 (37.93%)	
≥8	16 (12.50%)	3 (1.61%)	0	
Pathological stage				0.05
≤pT2	81 (63.28%)	139 (74.73%)	23 (79.31%)	
	47 (36.72%)	47 (25.27%)	6 (20.69%)	
Positive points (tumor extent)				
≤26	44 (37.93%)	98 (55.06%)	15 (60.00%)	0.009
	72 (62.07%)	80 (44.94%)	10 (40.00%)	
Positive points (median/min max)	46.70 (1.00–33.50)	29.76 (0–222)	33.32 (1.0–152)	<0.001
“Insignificant” tumors*				<0.001
Yes	0 (0.00%)	22 (12.43%)	3 (12.50%)	
No	116 (100.00%)	155 (87.57%)	21 (87.50%)	
Positive surgical margin				<0.001
No	51 (40.16%)	114 (61.29%)	20 (68.97%)	
Yes	76 (59.84%)	72 (38.71%)	9 (31.03%)	
Extra prostatic extension				0.043
No	81 (63.78%)	141 (75.81%)	23 (79.31%)	
Yes	46 (36.22%)	45 (24.19%)	6 (20.69%)	
Seminal vesicle invasion				0.003
No	103 (82.40%)	174 (94.57%)	25 (86.21%)	
Yes	22 (17.60%)	10 (5.43%)	4 (13.79%)	

*Based on classification of Billis et al. [6].

**Table 2:** Univariate logistic regression analysis to preoperatively predict Gleason discordance.

Variable	Categories	*P* value	Odds ratio	CI 95% OR
Race	White*	—	1.00	—
	Black	0.281	1.38	0.77–2.49
Age	Years	0.049	1.035	1.001–1.070
Clinical stage	T1c*	—	1.00	—
	T2a	0.030	1.79	1.06–3.03
	T2b	0.438	0.75	0.37–1.55
	T2c	0.128	5.11	0.62–41.81
Clinical stage (T1c × T2)	T1c*	—	1.00	—
	T2	0.092	1.49	0.94–2.37
PSA	ng/dL	<0.001	1.080	1.032–1.130
PSA (> or <10 ng/ml)	<10 ng/mL*	—	1.00	—
	>10 ng/mL	<0.001	2.48	1.50–4.11
Prostate weight	g	0.005	0.984	0.973–0.995
PSA density	0.1 unity	<0.001	1.713	1.394–2.104
Biopsy Gleason score	≤6*	—	1.00	—
	7	<0.001	19.13	7.50–48.80

*Categories of reference; CI: confidence interval; OR: odds ratio.

**Table 3:** Significant variables on multivariate logistic regression analysis to preoperatively predict Gleason discordance.

Variables	Categories	*P* value	Odds ratio	CI 95% OR
Biopsy Gleason score	≤6*	—	1.00	—
	7	<0.001	21.04	7.77–56.99
Prostate weight	g	<0.001	0.976	0.962–0.990
PSA	ng/dL	0.003	1.102	1.033–1.175
PSA density	0.1 unity	<0.001	1.720	1.400–2.113

*Category of reference; OR: odds ratio.

## References

[1] Pinthus JH, Witkos M, Fleshner NE (2006). Prostate cancers scored as Gleason 6 on prostate biopsy are frequently Gleason 7 tumors at radical prostatectomy: implication on outcome. *Journal of Urology*.

[2] King CR, McNeal JE, Gill H, Presti JC (2004). Extended prostate biopsy scheme improves reliability of Gleason grading: implications for radiotherapy patients. *International Journal of Radiation Oncology Biology Physics*.

[3] Chun FK, Steuber T, Erbersdobler A (2006). Development and internal validation of a nomogram predicting the probability of prostate cancer Gleason sum upgrading between biopsy and radical prostatectomy pathology. *European Urology*.

[4] Gonzalgo ML, Bastian PJ, Mangold LA (2006). Relationship between primary Gleason pattern on needle biopsy and clinicopathologic outcomes among men with Gleason score 7 adenocarcinoma of the prostate. *Urology*.

[5] Kvåle R, Møller B, Wahlqvist R (2009). Concordance between Gleason scores of needle biopsies and radical prostatectomy specimens: a population-based study. *BJU International*.

[6] Billis A, Magna LA, Ferreira U (2003). Correlation between tumor extent in radical prostatectomies and preoperative PSA, histological grade, surgical margins, and extraprostatic extension: application of a new practical method for tumor extent evaluation. *International Braz J Urol*.

[7] Epstein JI, Allsbrook WC, Amin MB (2005). The 2005 International Society of Urological Pathology (ISUP) consensus conference on Gleason grading of prostatic carcinoma. *American Journal of Surgical Pathology*.

[8] Cookson MS, Aus G, Burnett AL (2007). Variation in the definition of biochemical recurrence in patients treated for localized prostate cancer: the American Urological Association Prostate guidelines for localized prostate cancer update panel report and recommendations for a standard in the reporting of surgical outcomes. *Journal of Urology*.

[9] Colleselli D, Pelzer AE, Steiner E (2010). Upgrading of Gleason score 6 prostate cancers on biopsy after prostatectomy in the low and intermediate tPSA range. *Prostate Cancer and Prostatic Diseases*.

[10] Montironi R, Mazzucchelli R, Scarpelli M (2006). Prostate carcinoma II: prognostic factors in prostate needle biopsies. *BJU International*.

[11] Fitzsimons NJ, Presti JC, Kane CJ (2006). Is biopsy Gleason score independently associated with biochemical progression following radical prostatectomy after adjusting for pathological Gleason score?. *Journal of Urology*.

[12] Müntener M, Epstein JI, Hernandez DJ (2008). Prognostic significance of Gleason score discrepancies between needle biopsy and radical prostatectomy. *European Urology*.

[13] Serkin FB, Soderdahl DW, Cullen J, Chen Y, Hernandez J (2010). Patient risk stratification using Gleason score concordance and upgrading among men with prostate biopsy Gleason score 6 or 7. *Urologic Oncology*.

[14] Freedland SJ, Kane CJ, Amling CL, Aronson WJ, Terris MK, Presti JC (2007). Upgrading and downgrading of prostate needle biopsy specimens: risk factors and clinical implications. *Urology*.

[15] Sved PD, Gomez P, Manoharan M, Kim SS, Soloway MS (2004). Limitations of biopsy Gleason grade: implications for counseling patients with biopsy Gleason score 6 prostate cancer. *Journal of Urology*.

[16] Ozden C, Oztekin CV, Ugurlu O, Gokkaya S, Yaris M, Memis A (2009). Correlation between upgrading of prostate biopsy and biochemical failure and unfavorable pathology after radical prostatectomy. *Urologia Internationalis*.

[17] Hong SK, Han BK, Lee ST (2009). Prediction of Gleason score upgrading in low-risk prostate cancers diagnosed via multi (≥12)-core prostate biopsy. *World Journal of Urology*.

[18] Dong F, Jones JS, Stephenson AJ, Magi-Galluzzi C, Reuther AM, Klein EA (2008). Prostate cancer volume at biopsy predicts clinically significant upgrading. *Journal of Urology*.

[19] Liu JJ, Brooks JD, Ferrari M, Nolley R, Presti JC (2011). Small prostate size and high grade disease-biology or artifact?. *Journal of Urology*.

[20] Ngo TC, Conti SL, Shinghal R, Presti JC (2012). Prostate size does not predict high grade cancer. *Journal of Urology*.

[21] Rahmouni A, Yang A, Tempany CM (1992). Accuracy of in-vivo assessment of prostatic volume by MRI and transrectal ultrasonography. *Journal of Computer Assisted Tomography*.

[22] Varma M, Morgan JM (2010). The weight of the prostate gland is an excellent surrogate for gland volume. *Histopathology*.

