# Supplementary figures and images for: Higher Prostate Weight Is Inversely Associated with Gleason Score Upgrading in Radical Prostatectomy Specimens
Source: Adv Urol. 2013 Oct 31;2013:710421. doi: 10.1155/2013/710421 (PMC3833008; doi:10.1155/2013/710421)

**Supplementary Figure.** PSA density (≥ 0.263) determined disease free survival


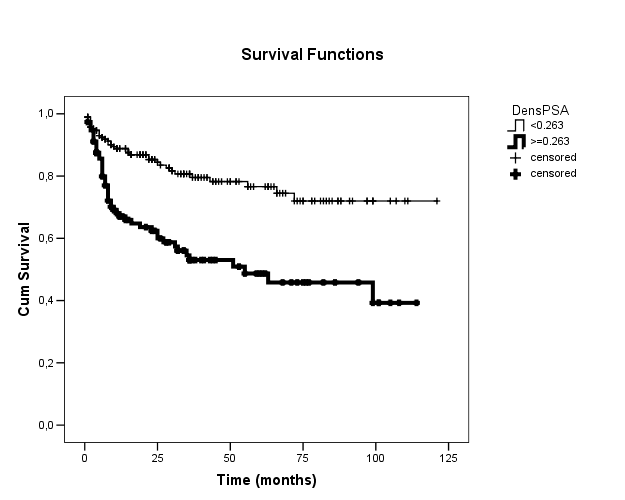


log-rank: χ2=22.76; GL=1; **p<0.001**

Supplement: Supplementary file 1 — Supplementary Figure: PSA density (≥ 0.263) determined disease free survival. [file 710421.f1.docx]
